# Multilevel correlates of childhood violence in refugee settings: findings from the Ethiopia humanitarian violence against children and youth survey

**DOI:** 10.1080/16549716.2026.2647656

**Published:** 2026-03-27

**Authors:** Yadeta Dessie, Bonnie Wandera, George Odwe, Francis Obare, Dagim Habteyesus, Stella Muthuri, Gloria Seruwagi, Ushuu Namarra, Abir Nur, Peter Kisaakye, Caroline W. Kabiru, Chi-Chi Undie, Yohannes Dibaba Wado

**Affiliations:** a African Population and Health Research Center, Nairobi, Kenya; b College of Health and Medical Sciences, Haramaya University, Harar, Ethiopia; c Population Council-Kenya, Nairobi, Kenya; d Population Council-Ethiopia, Addis Ababa, Ethiopia; e Yale School of Public Health, New Haven, CT, USA

**Keywords:** Socioecological model, children, RRS, HVACS, Ethiopia

## Abstract

**Background:**

Despite their right to protection, children in refugee settings face various forms of violence, including physical, sexual, and emotional violence.

**Objective:**

This study examined the factors associated with childhood violence (before turning 18) in refugee settings of Ethiopia, guided by the socioecological framework.

**Methods:**

The study used data from the 2024 Ethiopia Humanitarian Violence Against Children and Youth Survey (HVACS). This cross-sectional survey included females and males aged 13–24 years. We estimated a mixed-effects regression model to examine the correlates associated with experiencing violence in childhood in the refugee camps by taking into account camp-level clustering.

**Results:**

The study involved a total of 3473 respondents (1937 females and 1536 males) and revealed that about one in three (33.3%; 95% CI: 27.5, 39.6) had experienced childhood violence; the highest proportion 29.1% [23.1,35.9] reported experiencing physical violence, followed by emotional violence (12.4% [8.5,17.8]) and sexual violence (6.6% [5.3,8.1]). Correlates of experiencing childhood violence included being an orphan, having any form of disability, witnessing intimate partner violence against women, and having family members who were killed or died unnaturally. In contrast, households headed by women and children living in families with two or more rooms, had a lower likelihood of experiencing childhood violence. At the community level, witnessing violent attacks in the village was associated with a higher likelihood of experiencing childhood violence.

**Conclusions:**

Childhood violence is prevalent in refugee settings in Ethiopia and is associated with factors occurring at multiple levels, suggesting for individual-, household-, and community - level prevention and response strategies.

## Paper context

- **Main findings:** Childhood violence was more likely to happen among children who are orphaned, with disability, involved in intimate relationships, witnessed intimate violence against women at home, and exposed to past or recent violence.
- **Added knowledge:** The study generated peculiar attributes of childhood violence in the refugee contexts in Ethiopia, which provide new insights into prevention and response efforts towards childhood violence, that have not been adequately addressed in the humanitarian context. 
- **Global health impact for policy and action:** Given that about 40 million people are living in refugee settings, of which about half of them are children, the evidence from this study is important for guiding the design of effective approaches to prevent and respond to childhood violence and is relevant for addressing different global and regional initiatives fighting against this pervasive problem.

## Background

Childhood violence entails any forms of abuse or maltreatment of individuals under the age of 18—including physical, sexual, and emotional violence. The problem remains one of the most pressing global public health issues, and every year, an estimated 1 billion children, or about half of the world’s youth population, experience some form of violence which translates to about 110,000 children being affected every hour [1,2]. Multiple factors that contribute to the occurrence of violence against children have been documented, particularly within developmental settings. The socioecological model posits that childhood violence results from the interaction of factors across multiple levels, including the individual, family, community, and societal level. The individual-level factors include characteristics of the child, such as age, gender, and educational status; family/household factors include socio-demographic composition and economic conditions of households as well as parenting style, while community-level factors include the local environment with which the child primarily interacts [3–5].

Childhood violence occurs everywhere, but it is more common in humanitarian and unstable environments such as war zones, disaster areas, and refugee camps [6]. It is estimated that a quarter of the world’s population lives in fragile settings, with a significant number forcibly displaced as refugees [7]. A substantial proportion (75%) reside in low- and middle-income countries, with the vast majority being in sub-Saharan Africa [8]. It is, for instance, estimated that there are about 6 million refugees in the East and Horn of Africa and the Great Lakes region, with the majority being hosted in Uganda, Ethiopia, and Kenya [9]. Two-in-five of the displaced population in the region are children under 18 years of age [10]. Ethiopia hosts about 1 million refugees and asylum seekers of whom about half are children [11].

Evidence from the first ever Humanitarian Violence Against Children and Youth Survey (HVACS) in Uganda’s refugee settlements showed that half of males and 43% of females aged 18–24 years experienced any type of violence (sexual, physical, or emotional) before age 18, while 5% of males and 4% of females experienced all the three types of violence in their childhood [12]. Among 13–17-year-olds, 65% of males and 49% of females reported ever experiencing any type of violence [12]. A recent study in the same country that involved school-based screening showed even a higher prevalence of lifetime sexual violence among females (82%) and among males (83%) [13]. A systematic review of the prevalence of violence against children in the humanitarian contexts showed the occurrence of physical violence ranging from 9.0% to 92.0% and sexual violence ranging from 4% to 72% among different sub-groups of children [14]. In Ethiopia, a recent nationally representative violence against children survey showed that one in 10 females (10.5%) and 2.9% of males experienced lifetime sexual violence, two in five females (40.2%) and one in two males (54.6%) experienced lifetime physical violence, and about one in five females (20.5%) and one in 10 males (13.5%) experienced lifetime emotional violence [15].

Evidence shows that married youth are at higher risk of experiencing violence compared to those who are not married, while those who live with their biological mothers are less likely to have experienced any form of violence [16]. Material hardship, including difficulties affording basic needs like housing, food, utilities, and medical care, as well as families reporting housing instability (example, difficulty paying rent, eviction) and food insecurity, are more likely to be associated with child maltreatment and abuse [17,18]. Moreover, household living conditions and community- level factors such as prevalence of violence and crimes are also associated with childhood abuse [19,20]. Evidence from the Young Lives Matter study using social ecological approaches showed several factors that operate at the individual level, including discriminatory social and gender norms such as class, ethnicity, and gender; overcrowded situations and lack of protection associated with poverty and structural and institutional factors are associated with a high risk of violence [3]. Furthermore, a previous study using data from a national violence against children and youth survey found that better-off households, higher levels of educational attainment, and children in a marriage-likerelationship had the highest risk of experiencing violence [21].

The evidence suggests that as much as the different factors operating at individual, household, community, and societal levels persist in different contexts in sub-Saharan Africa, the much anticipated agenda by the African Union to end any form of childhood violence in Africa by 2040 may not be realized unless innovative and context-oriented interventions are designed to prevent and respond to violence against children [22]. This suggests a need for context-specific evidence on the extent of childhood violence and the factors associated with the occurrence of violence at different levels (individual, household, community, and society), particularly in fragile contexts. However, rigorous evidence on childhood violence in fragile contexts (such as humanitarian and refugee contexts) in the region is sparse, with much of the existing literature focusing on development (non-fragile) settings. Additionally, available evidence often utilizes narrow definitions of childhood violence, overlooking certain aspects and failing to consider multi-level factors [4,23,24]. Our study aims to fill these gaps by examining childhood violence using a broader definition and standard methodology within refugee settings in Ethiopia. Guided by the socioecological model, we examined the individual, family/household, and community-level factors associated with childhood violence among individuals aged 13–24 years living in refugee camps in Ethiopia, accounting for potential unobservable camp-level factors.

## Methods and procedures

### Study design and setting

Data are from the Ethiopia Humanitarian Violence Against Children and Youth Survey (HVACS), conducted from December 2023 to April 2024 [25]. The Ethiopia HVACS was a representative cross-sectional household survey of 13 to 24-year-old females and males living in refugee camps in the country. Ethiopia has a long history of receiving asylum seekers and hosting refugees. It is home to about 1 million refugees from more than 26 countries who have settled in camps across different regions (add reference here). Refugees from South Sudan, Somalia, Eritrea, and Sudan make up the largest proportion [26]. The study was conducted in 20 out of 23 refugee camps in Gambella, Beninshangul-Gumuz, Somali-Jigjiga, Somali-Melkadida, and Afar, while three camps were excluded due to security concerns at the time of data collection.

## Study sampling

The survey was conducted using the standard Violence Against Children and Youth (VACS) methodology. VACS is a standardized methodology that has been applied in over 20 low- and middle-income countries to obtain population-based national estimates (and often sub-national) prevalence estimates of violence against children and youth. It uses a standardized sampling approach and involves interviews with 13–24-year-old females and males on their past and current experiences of violence. Aggregated and weighted data provide estimates presented in VACS reports. VACS can be successfully adapted to a humanitarian context to provide estimates of violence against children and youth affected by humanitarian crises. A population living in a humanitarian context can be identified for VACS implementation without sampling of the host population. For example, in the context of refugee camps, a representative sample can be drawn only from within the camps [27]. This method was adapted and successfully applied in Uganda’s refugee settlements in the first-ever VACS conducted in a humanitarian setting [12]. The Ethiopia HVACS used the administrative units set by the Refugees and Returnees Service (RRS) – a governmental agency responsible for refugee affairs in Ethiopia – as the sampling unit. The survey employed a three-stage cluster sampling design-, by randomly selecting zones in the respective camps for both female and male samples. The survey used a split-sample approach, with female and male samples drawn from separate zones to protect confidentiality and minimize the risk of retaliation by preventing contact between survivors and potential opposite-sex perpetrators. In the first stage, 82 zones (41 for female and 41 for male interviews) were randomly sampled from the list of 158 zones provided by RRS. The second stage involved a sample of households from each zone based on probability proportional to size (PPS) [25]. The number of sampled households in each zone was based on the proportion of households in the zone relative to the total number of households in all selected zones, determined separately for the female and male samples. In the third stage, one eligible 13–24-year-old participant was randomly selected from each sampled household and provided assent/consent to participate in the survey. Finally, a total of 3473 children and young people (1937 females and 1536 males) were included in the analysis.

## Data collection

Data were collected electronically using a standardized questionnaire programmed in Open Data Kit (ODK) and administered through Android-enabled tablets. The interviews were conducted in safe, private locations at a secure distance from other household members, either within an appropriate area of the home or in the yard or under a tree in the compound. The HVACS questionnaire included a household module and individual modules (male and female) adapted for humanitarian settings. The household module was administered to heads of households, and the individual module was administered to eligible 13–24-year-old participants. The data collection tools were translated from English to seven local languages that are spoken in the sampled camps, which include Arabic (Juba Arabic), Anuak (Anywaa), Nuer, Somali, Amharic, Tigiringa, and Afar. The interviews were conducted in the respondents’ preferred languages, which were Nuer and Anuak in Gambella, Tigiringa in Afar, Somali in Jigjiga and Melkadida and Arabic in Beninshangul-Gumuz. Back translations were also made to check the consistency of the translated tools. Female interviewers conducted the interviews in female zones, while male interviewers conducted interviews in the male zones. The data collection team included research assistants and team leaders who received comprehensive training on survey protocol, study tools, ethical aspects of research, and the electronic data collection system. To ensure the data quality, the survey consent and questionnaire tools were piloted. Field supervision was provided by a total of 22 team leaders, each managing two or three research assistants and 5 regional field coordinators assigned to each region. The team leaders were responsible for helping research assistants identify sampled households and eligible participants. They validated the collected data at the end of each day before it was uploaded to the server. Regional coordinators held daily meetings with team leaders – either in person or virtually – to monitor data quality. Moreover, the data collection process was closely monitored by two research fellows who held daily debriefings with field teams. At a higher level, study implementation was monitored by the investigating team from both national and international institutions, who conducted debriefings with in-country research teams on a weekly basis and more frequently when deemed necessary.

## Measurements

### Outcome variable

#### Childhood violence

This was assessed from three dimensions. The first is sexual violence, which referred to a child’s experience with completed or attempted non-consensual sex acts, as well as unwanted touches. The second dimension is physical violence, which included any physical acts done by current or former intimate partners, peers, parents, adult caregivers, other adult relatives, or community members like teachers, police, employers, religious leaders, neighbors, or unfamiliar adults to harm the participant. The third type is emotional violence, measured by any reported experience of emotional harm caused by parents, adult caregivers, other adult relatives, intimate partners, or peers. Questions on these forms of violence were asked to individuals aged 13–17 years about whether they had ever experienced any of these forms of violence, while those aged 18–24 years were asked about their experiences of violence before the age of 18. A detailed description is provided in (Supplement 1). Participants who reported experiencing any of the three types of violence were categorized as ‘having experienced violence’, and those who did not were categorized as ‘having not experienced any form of violence’. This was guided by past research, which indicates that different forms of violence often occur concurrently and that polyvictimization is prevalent [28,29].

### Independent variables

#### Individual-level correlates

These included the respondent’s sex, categorized as male or female; age, classified as ’13–17“ and ”18–24’ years; education level, categorized as ‘primary and lower’ and ‘secondary and above’; relationship status, categorized as ‘ever married or have had a boyfriend/girlfriend and ‘otherwise’; orphanhood status, categorized as ‘orphaned’ (loss of one or both parents) and ‘not orphaned’. Disability status was assessed using the Washington Group Short Set on Functioning, which assesses difficulties in basic activities such as seeing, hearing, walking, cognition, self-care, and communication [30]. Respondents were categorized as having a disability if they reported difficulty in at least one domain, and as having no disability if none were reported.

#### Family/household level correlates

These variables included whether the household head was male or female, the number of living rooms in the household (one, two, three, or more), and the household wealth index. The wealth index was generated using principal component analysis (PCA) from various assets and amenities including toilet facilities (type and way of using them), cooking facilities, housing structure (wall, floor, and roof), number of rooms in a household, ownership of different modes of transport, ownership of functional electronic materials, and ownership of agricultural land and household livestock and their quantities. The first component of the wealth index was categorized into quartiles as lowest, second, third, and fourth. Witnessing intimate partner violence was measured by asking participants how many times (never, once, a few times, or many times) they saw or heard their mother or stepmother being punched, kicked, or beaten by their father or stepfather at any time in their lives (for 13- to 17-year-olds) and before turning 18 (for 18- to 24-year-olds). Responses were categorized as ‘1’ for those who reported witnessing violence at least once and ‘0’ otherwise. Household food insecurity was measured by the question, ‘How often during the last 12 months did you have problems getting enough food for the household?’ Responses were classified as never/seldom, frequently/always, or not reported. Concerns about having enough money for food were examined by the question, ‘How often during the last 12 months did you have problems getting enough food for the household? Would you say all the time, most of the time, sometimes, seldom, or never?’ Responses were coded as ‘yes’ if the participant reported any level of concern and ‘no’ if they answered ‘never.’ Participants were asked if any family members had died unnaturally, with responses categorized as ‘yes’ or ‘no.’

#### Community-level correlate

This was assessed by asking participants the question, ‘Outside of your shelter/home and family environment, how many times did you see anyone get attacked? Would you say never, once, or more than once?’ This was categorized as ‘yes’ if they reported witness and ‘no’ if they never witnessed.

## Data analysis

STATA ® version 17 was used for the analysis, which accounted for the complex survey design by applying weights to the estimates generated. The weights were calculated as the product of the inverse of the probability of sampling zones, households within zones, and individuals within households, adjusted for non-response at the household and individual levels, as well as the population of children and young people ages 13–24 years living in the camps, as provided by the RRS. We estimated a multilevel mixed-effects model that considered camps as the cluster. Thus, in estimating the model, we first ran a bivariate mixed-effects model that considered a single variable correlate, taking into account camp-level clustering. We have checked the adequacy of cluster size and cluster members to run the identified model, and they were within the acceptable state. We then estimated four models first without any correlates as null model (Model-I), and then by considering individual-level correlates (Model-II) which included sex, age in years, educational status, orphan status, history of having partner, disability status, and household- and societal-level correlates (Model – III) consisting of indicators of witnessing violence, household headship, number of rooms in household, experience of hunger in the last 12 months, and having a family member killed/dying unnaturally. The final model (Model-IV) included the variables from both Model II and Model III, and adjusted for clustering at the camp level. The intra-cluster correlation was estimated to examine the extent of clustering at the camp level. We conducted the Akaike Information Criterion (AIC), Bayesian Information Criterion (BIC), and likelihood ratio tests to determine the model fitness. We compared the values for both AIC and BIC and considered the one with the lowest value as the best-fit model. A *p*-value of less than 0.05 with 95% confidence intervals was used to determine the level of significance of the association.

## Ethical considerations

The Ethiopia HVACS was approved by the Population Council Institutional Review Board (Protocol 986) and the Ethiopian Public Health Association (EPHA) Institutional Review Board (EPHA/OG/789/23). The RRS provided administrative authorization to enter the camps. The study followed WHO’s Ethical and Safety Recommendations for Researching, Documenting, and Monitoring Sexual Violence in Emergencies [31], and Helsinki Declaration outlining ethical principles for medical research involving human participants [32]. All participants provided verbal informed consent to participate in the survey. Given the sensitive nature of the research, the waiver of signed informed consent by the participant aligned with our efforts to assure participants about the protection of confidentiality and privacy, as well as to adhere to WHO guidelines on the subject. Participants aged 18–24 years and emancipated minors aged 13–17 (participants who had assumed adult roles and responsibilities, including household headship, marriage, and/or procreation) provided individual consent. For dependent participants aged 13–17 years, interviewers first obtained permission from parents or primary caregivers to talk to the eligible participant before obtaining assent from the participants. A response plan that involved attaching case workers from United Nation Higher Commissioner for Refugee’s (UNHCR’s) implementing partners to the data collection teams was also built into the survey to ensure that participants in need of counseling received this immediately at the household level during or following the interview, or received a supported referral for such counseling at a service location after the interview.

## Results

### Socio-demographic characteristics

Of 3473 participants who completed the survey, 51.3% (*n* = 1536) were males, 51.9% (*n* = 1850) were aged 13–17 years, 20.1% (*n* = 605) were orphaned, and 14.6% (*n* = 439) reported some form of disability. About half (46.0%, *n* = 1560) had an intimate partner (married or boyfriend/girlfriend). Most households were headed by females (83.4%, *n* = 2656), and about two out of five respondents (40.0%, *n* = 1532) reported housing structures with two rooms. About one-sixth (15.3%,*n* = 499) reported witnessing their community being violently attacked at different times (Table 1).

**Table 1.** Demographic characteristics of participants, Ethiopia HVACS 2024.

Characteristics	N (3473)	Weighted %
**Sex**		
Female	1937	48.7
Male	1536	51.3
**Age in years**		
13–17	1850	51.9
18–24	1623	48.1
**Educational status**		
Primary and less	2721	75.3
Secondary and above	752	24.7
**Orphanhood status**		
Not orphaned	2868	79.9
Orphaned	605	20.1
**History of partnership**		
Never had a partner	1913	54.0
Ever had partner	1560	46.0
**Has any form of disability**		
No	3034	85.4
Yes	439	14.6
**Sex of head of household**		
Male	775	16.6
Female	2656	83.4
**Age of household head (in years)**		
≤35	1196	34.3
36–45	1225	38.6
≥46	1010	27.1
**Number of rooms in household**		
One	1165	34.5
Two	1532	40.8
Three and more	734	24.7
**Household wealth index**		
Lowest quantile	660	19.5
Second quantile	681	18.5
Third quantile	692	20.5
Fourth quantile	713	20.0
Highest quantile	777	21.4
**Experience of hunger in the last 12 months**		
Never/seldom	159	4.5
Frequently	2271	71.2
Always	845	20.4
Response not collected	198	3.9
**Worried or stressed out about having enough** **money to pay for meals**		
No	3294	95.3
Yes	179	4.8
**History of village attack**		
No	2974	84.7
Yes	499	15.3
**Family members killed/died unnaturally**		
No	3240	90.4
Yes	233	9.7

### Childhood violence

**Figure 1.**
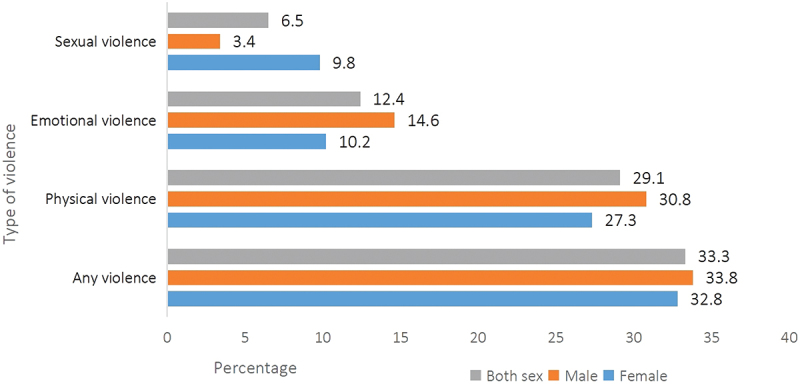
Prevalence of childhood violence in refugee settings, Ethiopia HVACS, 2024.

About one-in-three of the participants (33.3%; 95% CI: 27.5, 39.6) reported ever experiencing childhood violence. All weighted, physical violence was the most commonly reported form of childhood violence, experienced by 29.1% of the participants [23.1,35.9], followed by emotional violence (12.4% [8.5,17.8]) and sexual violence (6.6% [5.3, 8.1]) (Figure 1).

### Multilevel correlates of childhood violence

The bivariate mixed effects model showed that older age (crude odds ratio (COR)=0.56; 95% CI: 0.48, 0.65); higher educational level (COR = 0.61, 95% CI: 0.51, 0.74); history of intimate partnership (marriage or having a boyfriend/girlfriend) (COR = 1.20; 95% CI: 1.04, 1.39], disability (COR = 1.20, 95% CI: 1.04, 1.39), orphanhood status (COR = 1.46; 95% CI: 1.21. 1.77), households headed by females (COR = 0.71; 95% CI: 0.59, 0.86), number of rooms, either two (COR = 0.77; 95% CI: 0.64, 0.92) and three (COR = 0.62; 95% CI: 0.50, 0.78), always experiencing hunger (COR = 4.47; 95% CI: 3.83,5.23), having a close family member killed or who died unnaturally (COR = 2.52; 95% CI: 2.05, 3.09), and prevalence of community-level violence (COR = 3.08; 95% CI: 2.30, 4.14) were significantly associated with the likelihood of experiencing childhood violence (Table 2). In the building, the multilevel model, at first we ran a null model, is without adding a variable into it except the cluster effect. The second, displays consisted variables that include sex, age in years, educational status, orphan status history of having partner and disability status, and the third model included women intimate violence witnessing, household headship, household room possession, experience of hunger in the last 12 months, and family members killed/died unnaturally. In the final model that controlled for individual, family/household, and community-level factors, showed that several correlates were significantly associated with the odds of experiencing childhood violence. In particular, being an orphan (adjusted odds ratio (AOR) = 1.24; 95% CI: 1.00, 1.54), being married or having a boyfriend/girlfriend (AOR = 1.65 [1.36,2.02]), having any form of disability (AOR = 1.69; 95% CI: 1.32,2.14), witnessing violence at home (AOR = 4.18; 95% CI: 3.54, 4.95), and having a family member who was killed or died unnaturally (AOR = 1.96; 95% CI: 1.39, 2.77) were significantly associated with higher odds of experiencing childhood violence. Children in households headed by females (AOR = 0.68; 95% CI: 0.55, 0.84), those in households with two living rooms (AOR = 0.67; 95%: 0.55, 0.82) and those in households with three or more living rooms (AOR = 0.59; 95% CI: 0.46, 0.76) had significantly lower odds of experiencing childhood violence. At the community level, witnessing violent attacks in the community was significantly associated with higher odds of experiencing childhood violence (AOR = 2.08; 95% CI: 1.65, 2.63). The intra-cluster (ICC) correlation of the null model was 10.6%, while the final model produced an ICC of 9.3%, which indicats the model is well specified. The level ICC is acceptable as long as the model converged and, importantly, for the critical outcome, which is a very sensitive human rights violation where even a low ICC provides great insights for any camp-tailored intervention (Table 3).

**Table 2.** Results from bivariate analysis of correlates of childhood violence in refugee settings, Ethiopia HVACS 2024.

Variables	N(3473)	Experienced Violence			
			Weighted percentage with 95% CI	COR (95% CI)	*p*-value
**Sex**					
Female	1937	644	32.75 [26.46,39.74]	1	
Male	1536	550	33.79 [24.57,44.42]	1.11 (0.95, 1.29)	0.19
**Age in years**					
13–17	1850	749	37.11[30.41,44.35]	1	
18–24	1623	445	29.15[23.76,35.21]	0.56(0.48, 0.65)**	0.00
**Educational status**					
Primary and less	2721	997	34.98 [29.21,41.23]	1	
Secondary and above	752	197	28.10[20.64,37.00]	0.61(0.51, 0.74)**	0.00
**Orphanhood status**					
Not orphaned	2868	955	30.94[24.85,37.77]	1	
Orphaned	605	239	42.59[33.50,52.21]	1.46[1.21. 1.77]**	0.00
**History of partnership**					
Never had partner	1913	622	29.46 [23.40,36.35]	1	
Ever had partner	1560	572	37.76 [31.42,44.56]	1.20[1.04,1.39]*	0.01
**Has any form of disability**					
No	3034	975	31.75[25.79,38.37]	1	
Yes	439	219	42.30[29.80,55.86]	2.04[1.65, 2.54]**	0.00
**Sex of head of household**					
Male	775	332	44.47[34.92,54.45]	1	
Female	2656	852	31.18[25.92,36.98]	0.71[0.59, 0.86]**	0.00
**Number of rooms in household**					
One	1165	462	38.63[29.54,48.60]	1	
Two	1532	507	31.65[25.93,37.99]	0.77[0.64,0.92]**	0.00
Three and more	734	215	28.96[21.95,37.14]	0.62[0.50,0.78]**	0.00
**Household wealth index**					
Lowest quantile	660	220	32.09 [25.22,39.83]	1	
Second quantile	681	230	31.27 [24.28,39.24]	1.02[0.80.1.28]	0.89
Third quantile	692	242	33.67 [25.92,42.41]	1.07[0.85,1.35]	0.56
Fourth quantile	713	240	33.27 [27.07,40.10]	1.02[0.81,1.29]	0.86
Highest quantile	777	262	35.75 [27.95,44.39]	1.10[0.88,1.38]	0.41
**Witnessing violence at home**					
No	1,622	410	18.9 [15.1,23.4]	1	
Yes	657	784	51.5 [43.2,59.6]	4.47[3.83,5.23]*** [3.825,5.231]	0.00
**Experience of hunger in the last 12 months**					
Never/seldom	159	37	24.41[13.56,39.92]	1	
Frequently	2271	760	33.80[27.11,41.21]	1.34[0.91, 1.98]	0.14
Always	845	338	34.23[26.23,43.24]	1.84[1.22, 2.77]**	0.00
Response not collected	198	59	29.13[19.26,41.44]	1.20[0.73, 1.97]	0.46
**Worried or stressed out about having enough** **money to pay for meals**					
No	3294	1148	33.62[27.70,40.10]	1	
Yes	179	46	26.61[16.40,40.12]	0.80[0.56, 1.15]	0.23
**History of village attack**					
No	2974	951	30.45 [24.69,36.89]	1	
Yes	499	243	49.03 [39.63,58.50]	2.52 [2.05, 3.09]**	0.00
**Family members killed/died unnaturally**					
No	3240	1056	31.04[25.60,37.05]	1	
Yes	233	138	54.31 [42.51,65.64]	3.08[2.30, 4.14]**	0.00

Odds ratios; 95% confidence intervals in brackets* *p* < 0.05, ** *p* < 0.01, *** *p* < 0.001.

**Table 3.** Results from multivariable logistic regression examining correlates of experiencing childhood violence among children and youth in refugee settings, Ethiopia HVACS 2024.

		Model-II		Model-III		Final Model(Model-IV)	
Variables	Null Model(Model-I)	AOR	95% CI	AOR	95% CI	AOR	95% CI
**Sex**							
Female		1	1			1	1
Male		1.14	[0.98,1.34]			1.23*	[1.03,1.46]
**Age in years**							
13–17		1	1			1	1
18–24		0.39***	[0.33,0.48]			[0.30,0.45]	[0.29,0.45]
**Educational statu**s							
Primary and less		1	1			1	1
Secondary and above		0.68***	[0.56,0.83]			[0.58,0.90]	[0.58,0.90]
**Orphanhood status**							
Not orphaned		1	1			1	1
Orphaned		1.41***	[1.16,1.71]			1.24*	[1.00,1.54]
**History of having partner**							
Never had		1	1			1	**1**
Ever had		2.09***	[1.74,2.51]			1.65***	[1.36,2.02]
**Has any form of disability**							
No		1	1			1	1
Yes		2.04***	[1.63,2.55]			1.68***	[1.32,2.14]
**Witnessing violence at home**							
No				1		1	1
Yes				4.42***	[3.77,5.19]	4.18***	[3.54,4.95]
**Sex of household head**							
Male				1		1	1
Female				0.72**	[0.59,0.89]	0.684***	[0.55,0.84]
Not reported				0.596	[0.25,1.43]	0.413	[0.17,1.02]
**Number of rooms in household**							
One				1		1	1
Two				0.68***	[0.56, 0.82]	0.672***	[0.55,0.82]
Three and more				0.59***	[0.46,0.75]	0.594***	[0.46,0.76]
Not reported				-	–	–	–
**Experience of hunger in the last 12 months**							
Never/seldom				1	1	1	1
Frequently				0.91	[0.60,1.39]	0.94	[0.62,1.44]
Always				1.21	[0.78,1.87]	1.23	[0.78,1.92]
Not reported				0.81	[0.47,1.41]	1.17	[0.66,2.07]
**Family members killed/died unnaturally**							
No				1	1	1	1
Yes				2.57***	[1.88,3.51]	1.96***	[1.39,2.77]
**Witnessing village attack**							
No						1	
Yes						2.08***	[1.65,2.63]
Camp level variances	1.48**[1.11,1.96]	1.46**[1.11,1.94]		1.18*	[1.03,1.36]	1.20*	[1.03,1.41]
AIC	4349.70	4155.56		3903.69		3743.25	
BIC	4337.39	4204.78		3971.38		3853.99	
ICC%	10.56	10.36		8.70		9.26	

AIC: Akaike Information Criterion; BIC: Bayesian Information Criterion,and ICC: Intracluster correlation. Odds ratios; 95% confidence intervals in brackets* *p* < 0.05, ** *p* < 0.01, *** *p* < 0.001.

## Discussion

The study findings revealed that childhood violence is pervasive in refugee settings in Ethiopia. Factors associated with higher likelihood of experiencing childhood violence included being orphaned, having a history of marriage or intimate partnerships, living with a disability, and witnessing intimate partner violence at home. Additionally, witnessing violent attacks in one’s community and having family members who died unnaturally or were killed were significantly associated with a higher likelihood of experiencing childhood violence. In contrast, living in a female-headed household or a home with multiple rooms was associated with a lower likelihood of experiencing childhood violence.

Approximately one-third of respondents reported experiencing any form of childhood violence, which was slightly lower than the estimate from the Uganda Humanitarian Violence Against Children Survey (HVACS) and significantly lower than results from school-based sexual violence screenings in the same country [13]. While cultural and contextual differences may explain the modest variation between the two HVACS, the significant disparity with the school-based screening likely stems from differences in design and technique. Specifically, the school-based study utilized sensitization and a two-stage interview process for non-disclosing respondents, whereas the current study followed a community-based design with a single interview approach. Importantly, the prevalence of violence in refugee settings in Ethiopia aligns closely with estimates from the Ethiopia national VACS [15]. Notably, both the national VACS and HVACS were conducted around the same timeframe, providing complementary evidence to inform strategies to prevent or respond to violence against children in humanitarian and non-humanitarian settings. Future programmatic considerations would benefit from integrating evidence from the two surveys (national VACS and HVACS), particularly as the Ethiopian government pursues the refugees integrations into the general population [33], which ultimately helps to accelerate strategies for ending childhood violence on the continent by 2040 [22].

Our study showed that orphaned children were at about two times higher risk of experiencing childhood violence compared to those who were not. This is a finding consistent with prior evidence, as these sub-groups of the population are largely subjected to violence [34]. Various factors may explain the elevated risk of experiencing childhood violence among orphans. One of them could be diminished protection following the loss of a parent, potentially exposing the children to various perpetrators from within or outside of their families. It also could be from the repercussions that emanate from integrating orphaned children into extended families – a common practice after the loss of a parent – which may expose them to further violence in many ways [35]. Given the humanitarian context where some of the established norms and cultures for protecting children from violence are eroded, there is a need for particular consideration of the needs of children who are at higher risk of experiencing compounded crises such as those who are orphaned and living in a humanitarian setting.

Similar to what was reported from Uganda refugee settings [36], this study revealed that disability was significantly associated with the likelihood of experiencing violence. This association has also been consistently reported in non-humanitarian settings [37]. These could be due to the increased dependence of such children on caregivers and others around them. As a result, they are vulnerable to exploitation and maltreatment, as they may not be in a position to fend off incidents perpetrated by caregivers or the people around them. Furthermore, the poor and negative societal attitudes toward children with disabilities could also contribute to their high risk of being exposed to such violence [38]. This is particularly critical for those in humanitarian settings, which require coordinated and holistic interventions that focus on the life course of such children [39].

We found that those who reported being married or having a boyfriend/girlfriend were significantly more likely to experience childhood violence. A large body of evidence has shown that violence happens between intimate or romantic partners [40], particularly in the context of early marriage and among children and young people, where dating violence is prevalent [41,42]. These types of marriages/partnerships often occur at a very young age, in contexts of severe resource scarcity and insecurity, which may contribute to increased incidents of violence. The consequences of such violence within the relationship are far-reaching, often resulting in the onset or worsening of various immediate and long-term mental health issues and risky health behaviors, including suicide attempts and antisocial behavior [42,43]. Interventions targeting these individuals can include individual counseling or group-based training [44]. In addition, interventions focusing on supporting healthy relationships through various life skills education can be effective in preventing and responding to violence that could occur in contexts of early marriage [45].

Witnessing household-level intimate violence was significantly associated with the likelihood of experiencing childhood violence. This finding was consistent with that of the study in Uganda, both in non-humanitarian and refugee settings [46,47]. Another previous study also showed similar finding revealing the multidimensional effect of witnessing intimate violence on childhood experience of maltreatment and related behaviors [48]. This could occur due to unhealthy relationship in the household, which significantly contributes to emotional and psychological harm. The children might also be subjected to violence meted out to women in the household. Furthermore, the children might normalize such incidents, which may influence them to perpetrate violence in the course of their lives.

Contrary to what might be expected regarding household headship and exposure to violence [49,50], our study found a lower likelihood of experiencing childhood violence in female- compared to male-headed households. Research on parenting styles indicates that mothers tend to be more engaged in providing their families with proper knowledge and life skills [51], which may explain this finding. Furthermore, this could also indicate that female empowerment initiatives have positively impacted women, allowing them to take on leadership roles which have a broader effect, including taking action to prevent childhood violence [52].

In this study, owning multiple living rooms was significantly associated with a lower likelihood of experiencing childhood violence. The association between household overcrowding and child maltreatment has been established in previous studies, showing a 23–46% increase in child sexual abuse and a 40% higher risk of substantiated sexual abuse in overcrowded settings [53]. Likewise, the implications of housing conditions for child maltreatment were highlighted in the recent review conducted in developed settings [54]. Substandard housing is often associated with unstable living conditions, which might create stressful and tense environments within or between families, contributing to violence. Large family sizes are common in refugee settings, exacerbating the situation further, which coupled with resource constraints, may heighten tensions that require conscious consideration in improving the living conditions through the implementation of such initiatives as the Sustainable Shelter and Settlement Response Roadmap [55]

At the community level, we examined whether witnessing violence at the community level is correlated with experiencing childhood violence, and found a statistically significant positive association. This aligns with previous research showing that children living in unsecured neighborhoods are particularly at risk of experiencing violence [56,57]. This is worthing serious consideration, as maintaining security should be a top priority given that refugee settings are intended to serve as safe haven for those displaced from their homes of origin.

## Strengths and limitations

This is the first study in the country to comprehensively examine the correlates of childhood violence using a large sample of children living in refugee settings in Ethiopia. This large sample enabled a multilevel analysis that accounted for camp-level clustering of childhood violence. However, the cross-sectional nature of the data does not allow us to infer causality from the observed associations. Results are likely to be affected by telescoping bias. Backward telescoping is the inaccurate recall of the timing of violent events, where respondents remember incidents as having happened longer ago than they actually did. This issue is particularly relevant for participants aged 18–24 years, who may have under-reported childhood experiences of violence. A related issue is the sensitive nature of questions on violence, which may be subjected to social desirability bias. Furthermore, we combined the three types of violence in our analysis as disaggregated analysis was not possible due to the small magnitude of each type of violence; however, this may mask the distinct factors associated with each type of violence. Finally, our modeling approach did not involve a sex-aggregated analysis as it greatly diminished the statistical power, especially when considering the camp-level clustering. However, to reduce any differential associations, we controlled for sex in the analysis.

## Conclusions

This is the first comprehensive study to investigate the multilevel correlates of childhood violence in refugee settings in Ethiopia, revealing significant risk factors that warrant targeted prevention and response efforts at different levels. Children who are orphaned, living with a disability, or involved in romantic relationships require tailored interventions. These should also address household-level factors such as livelihood, family size, and headship, as well as community-level conditions, particularly in areas where violence is prevalent.

## Supplementary Material

Supplementray1_STROBE.doc

Appendix1_Childhood violence descriptions.docx

## Data Availability

The data are available on reasonable request.
