# Supplementary material for: Multilevel correlates of childhood violence in refugee settings: findings from the Ethiopia humanitarian violence against children and youth survey
Source: Glob Health Action. 2026 Mar 27;19(1):2647656. doi: 10.1080/16549716.2026.2647656 (PMC13034712; doi:10.1080/16549716.2026.2647656)
Supplement: Supplementray1_STROBE.doc [file ZGHA_A_2647656_SM4135.doc]

STROBE Statement—Checklist of items that should be included in reports of ***cross-sectional studies***

|  | Item No | Recommendation | Checked | Descriptions |  |
| --- | --- | --- | --- | --- | --- |
| **Title and abstract** | 1 | (*a*) Indicate the study’s design with a commonly used term in the title or the abstract | Yes | This was presented under the method section of the abstract. |  |
|  |  | (*b*) Provide in the abstract an informative and balanced summary of what was done and what was found | Yes | The abstract section summarized the study in the required layout. |  |
| Introduction | | |  |  |  |
| Background/rationale | 2 | Explain the scientific background and rationale for the investigation being reported | Yes | These were presented under the background part of the document. |  |
| Objectives | 3 | State specific objectives, including any prespecified hypotheses | Yes | These are stated in the last paragraph of the background. |  |
| Methods | | |  |  |  |
| Study design | 4 | Present key elements of study design early in the paper | Yes | This is indicated on the first page under the method section. |  |
| Setting | 5 | Describe the setting, locations, and relevant dates, including periods of recruitment, exposure, follow-up, and data collection | Yes | This is indicated on the first page under the method section on the first page. |  |
| Participants | 6 | (*a*) Give the eligibility criteria, and the sources and methods of selection of participants | Yes | These points are indicated on page 5 under the method section. |  |
| Variables | 7 | Clearly define all outcomes, exposures, predictors, potential confounders, and effect modifiers. Give diagnostic criteria, if applicable | Yes | These are all presented as in detail on page 6 and 7 under the method section. |  |
| Data sources/ measurement | 8* | For each variable of interest, give sources of data and details of methods of assessment (measurement). Describe comparability of assessment methods if there is more than one group | Yes | Presented in detail on page 6 and 7 under the sub-topic of ‘measurements’. |  |
| Bias | 9 | Describe any efforts to address potential sources of bias | Yes | These are indicated under the sampling and analysis sections. |  |
| Study size | 10 | Explain how the study size was arrived at | Yes | This is presented under the sampling method. |  |
| Quantitative variables | 11 | Explain how quantitative variables were handled in the analyses. If applicable, describe which groupings were chosen and why | Yes | These are indicated in detail under the measurements categorized as outcome and correlate variables. |  |
| Statistical methods | 12 | (*a*) Describe all statistical methods, including those used to control for confounding | Yes |  |  |
|  |  | (*b*) Describe any methods used to examine subgroups and interactions | NA | Not Applicable |  |
|  |  | (*c*) Explain how missing data were addressed | N/A | Not Applicable |  |
|  |  | (*d*) If applicable, describe analytical methods taking account of sampling strategy | Yes | Sample weighing was applied to consider the effect different level clusters. |  |
|  |  | (*e*) Describe any sensitivity analyses | NA | Not Applicable |  |
| Results | | |  |  |  |
| Participants | 13* | (a) Report numbers of individuals at each stage of study—eg numbers potentially eligible, examined for eligibility, confirmed eligible, included in the study, completing follow-up, and analysed | Yes | These are indicated under the sampling method substantiated with reference |  |
|  |  | (b) Give reasons for non-participation at each stage | NA | Not Applicable |  |
|  |  | (c) Consider use of a flow diagram | NA | Not Applicable |  |
| Descriptive data | 14* | (a) Give characteristics of study participants (eg demographic, clinical, social) and information on exposures and potential confounders | Yes | Presented under the result section |  |
|  |  | (b) Indicate number of participants with missing data for each variable of interest | Yes | Presented under the result section |  |
| Outcome data | 15* | Report numbers of outcome events or summary measures | Yes | Presented under the result section |  |
| Main results | 16 | (*a*) Give unadjusted estimates and, if applicable, confounder-adjusted estimates and their precision (eg, 95% confidence interval). Make clear which confounders were adjusted for and why they were included | Yes | Presented under the result section |  |
|  |  | (*b*) Report category boundaries when continuous variables were categorized | Yes | Presented under the result section |  |
|  |  | (*c*) If relevant, consider translating estimates of relative risk into absolute risk for a meaningful time period | Yes | Presented under the result section |  |
| Other analyses | 17 | Report other analyses done—eg analyses of subgroups and interactions, and sensitivity analyses | NA | Not Applicable |  |
| Discussion | | | Yes |  |  |
| Key results | 18 | Summarise key results with reference to study objectives | Yes | Presented on the first paragraph of the discussion section. |  |
| Limitations | 19 | Discuss limitations of the study, taking into account sources of potential bias or imprecision. Discuss both direction and magnitude of any potential bias | Yes | Limitations are included under the strength and limitations. |  |
| Interpretation | 20 | Give a cautious overall interpretation of results considering objectives, limitations, multiplicity of analyses, results from similar studies, and other relevant evidence | Yes | Interpretations are largely covered in the discussion sections. |  |
| Generalisability | 21 | Discuss the generalisability (external validity) of the study results | Yes | These indicated under the conclusion of the study largely and the discussion sections as well. |  |
| Other information | | | Yes |  |  |
| Funding | 22 | Give the source of funding and the role of the funders for the present study and, if applicable, for the original study on which the present article is based | Yes | This is indicated under the funding source sub-section. |  |

*Give information separately for exposed and unexposed groups.

Note: An Explanation and Elaboration article discusses each checklist item and gives methodological background and published examples of transparent reporting. The STROBE checklist is best used in conjunction with this article (freely available on the Web sites of PLoS Medicine at http://www.plosmedicine.org/, Annals of Internal Medicine at http://www.annals.org/, and Epidemiology at http://www.epidem.com/). Information on the STROBE Initiative is available at www.strobe-statement.org.
