# Supplementary material for: Multilevel correlates of childhood violence in refugee settings: findings from the Ethiopia humanitarian violence against children and youth survey
Source: Glob Health Action. 2026 Mar 27;19(1):2647656. doi: 10.1080/16549716.2026.2647656 (PMC13034712; doi:10.1080/16549716.2026.2647656)
Supplement: Appendix1_Childhood violence descriptions.docx [file ZGHA_A_2647656_SM4134.docx]

**Violence**: The World Health Organisation defines violence as “the intentional use of physical force or power, threatened or actual, against oneself, or another person, or against a group or community, which either results in or has a high likelihood of resulting in injury, death, psychological harm, mal-development or deprivation” (Krug et al., 2002).

**Sexual Violence:** Sexual violence encompasses a range of acts, including completed non-consensual sex acts, attempted nonconsensual sex acts, and abusive sexual contact. In the Ethiopia HVACS, questions were posed on four forms of sexual violence, including:

- *Unwanted Sexual Touching*: If anyone, male or female, ever touched the participant in a sexual way without their permission but did not try to force the participant to have sex. Touching in a sexual way without permission includes fondling, pinching, grabbing, or touching on or around the participant’s sexual body parts.
- *Unwanted Attempted Sex*: If anyone ever tried to make the participant have sex against their will but did not succeed (sex or sexual intercourse includes vaginal, oral, or anal sex). They might have tried to physically force the participant to have sex or they might have tried to pressure the participant to have sex through harassment or threats.
- *Pressured or Coerced Sex*: If anyone ever pressured the participant to have sex, through harassment or threats and did succeed in having sex with the participant.
- *Physically Forced Sex*: If anyone ever physically forced the participant to have sex and did succeed in having sex with the participant. In addition, questions were included about sex when a person was too drunk to give consent or say no. Although this is considered a form of sexual violence, it was not included in the sexual violence combined indicator because this question is new to the questionnaire and has not been fully tested or used in an African context.

**Physical Violence:** Participants were asked about physical acts of violence perpetrated by four types of potential perpetrators: 1) current or previous intimate partners, including a romantic partner, a boyfriend/girlfriend, or a spouse; 2) peers, including people the same age as the participant not including a boyfriend/girlfriend, spouse, or romantic partner, and might be people the participant may have known or not known including siblings, schoolmates, neighbours, or strangers; 3) parents, adult caregivers, or other adult relatives; and, 4) adults in the community such as teachers, police, employers, religious or community leaders, neighbours, or adults the participant did not know.

For each perpetrator type, participants were asked about four measures of physical violence: Has (1) an intimate partner; (2) a peer; (3) a parent, adult caregiver, or other adult relative; (4) an adult in the community ever:

- Slapped, pushed, shoved, shook, or intentionally threw something at the participant to hurt them.
- Punched, kicked, whipped, or beat the participant with an object.
- Choked, smothered, tried or attempted to drown, or burned the participant intentionally.
- Used or threatened the participant with a knife, gun or other weapon.

**Emotional Violence:** The behaviours measured for emotional violence varied according to the perpetrators. To assess emotional violence perpetrated by parents, adult caregivers or other adult relatives, participants were asked whether:

- The participant was told that they were not loved or did not deserve to be loved.
- The participant was told they (perpetrator) wished the participant had never been born or were dead.
- The participant was ridiculed or put down, for example, told that they were stupid or useless.

To assess emotional violence perpetrated by intimate partners, participants were asked if they had ever been treated the following way by a current or former romantic partner, boyfriend/girlfriend or spouse:

- - Insulted, humiliated, or made fun of in front of others.
  - Kept from having their own money.
  - Tried or kept from seeing or talking to their family or friends.
  - Kept track of by demanding to know where they were and what they were doing.
  - Threatened with intention to physically harm the participant.

To assess emotional violence by peers, participants were asked whether a person the participant’s own age had done the following in the past 12 months:

- Made the participant feel scared or feel really bad because they were calling the participant names, saying mean things to the participant, or saying they did not want them around.
- Told lies or spread rumours about the participant or tried to make others dislike the participant.
- Kept the participant out of things on purpose, excluded the participant from their group of friends, or completely ignored the participant.
